# Neurogranin-like immunoreactivity in the zebrafish brain during development

**DOI:** 10.1007/s00429-022-02550-6

**Published:** 2022-08-26

**Authors:** Anabel Alba-González, Julián Yáñez, Ramón Anadón, Mónica Folgueira

**Affiliations:** 1 Department of Biology, Faculty of Sciences, University of A Coruña, Campus da Zapateira, 15008-A Coruña, Spain; 2 Centro de Investigaciones Científicas Avanzadas (CICA), University of A Coruña, 15071-A Coruña, Spain; 3 Department of Functional Biology, Faculty of Biology, University of Santiago de Compostela, 15782 Santiago de Compostela, Spain

**Keywords:** Neurogranin, RC3, Immunohistochemistry, Brain development, Teleost, *Danio rerio*

## Abstract

Neurogranin (Nrgn) is a neural protein that is enriched in the cerebral cortex and is involved in synaptic plasticity via its interaction with calmodulin. Recently we reported its expression in the brain of the adult zebrafish (Alba-González et al. J Comp Neurol 530:1569–1587, 2022). In this study we analyze the development of Nrgn-like immunoreactivity (Nrgn-like-ir) in the brain and sensory structures of zebrafish embryos and larvae, using whole mounts and sections. First Nrgn-like positive neurons appeared by 2 day post-fertilization (dpf) in restricted areas of the brain, mostly in the pallium, epiphysis and hindbrain. Nrgn-like populations increased noticeably by 3 dpf, reaching an adult-like pattern in 6 dpf. Most Nrgn-like positive neurons were observed in the olfactory organ, retina (most ganglion cells, some amacrine and bipolar cells), pallium, lateral hypothalamus, thalamus, optic tectum, torus semicircularis, octavolateralis area, and viscerosensory column. Immunoreactivity was also observed in axonal tracts originating in Nrgn-like neuronal populations, namely, the projection of Nrgn-like immunopositive primary olfactory fibers to olfactory glomeruli, that of Nrgn-like positive pallial cells to the hypothalamus, the Nrgn-like-ir optic nerve to the pretectum and optic tectum, the Nrgn-like immunolabeled lateral hypothalamus to the contralateral region via the horizontal commissure, the octavolateralis area to the midbrain via the lateral lemniscus, and the viscerosensory column to the dorsal isthmus via the secondary gustatory tract. The late expression of Nrgn in zebrafish neurons is probably related to functional maturation of higher brain centers, as reported in the mammalian telencephalon. The analysis of Nrgn expression in the zebrafish brain suggests that it may be a useful marker for specific neuronal circuitries.

## Introduction

Neurogranin (Nrgn; also known as p17, RC3 and BICKS) is a small neural protein (Baudier et al. 1989, 1991; Watson et al. 1990; Coggins et al. 1993; Huang et al. 1993) that seems to regulate synaptic plasticity through its interaction with calmodulin and other proteins (Li et al. 2020; Zhong and Gerges 2020). Initially purified from bovine forebrain (named p17; Baudier et al. 1989, 1991), it was also identified as a cortex-enriched mRNA in rat brain (rat cortex-enriched cDNA clone 3 or RC3; Watson et al. 1990; see also Deloulme et al. 1991). Together with neuromodulin (GAP-43), PEP-19 (purkinje cell protein 4, pcp-4) and Igloo (Neel and Young, 1994; Gerendasy and Sutcliffe 1997; Gerendasy 1999), neurogranin is part of the so-called “calpacitin” family.

Neurogranin seems to regulate synaptic plasticity by favoring long-term potentiation (LTP) over long-term depression (LTD) (Fedorov et al. 1995; Ramakers et al. 1995, 1997; Chen et al. 1997; Pak et al. 2000; Huang et al. 2004; Lee 2006; Zhabotinsky et al. 2006; Zhong et al. 2009, 2011; Zhong and Gerges 2020). Neurogranin interacts with calmodulin through its highly conserved IQ domain (Baudier et al. 1989, 1991; Deloulme et al. 1991; Prichard et al. 1999), which also contains a specific site for protein kinase C (PKC) phosphorylation (Baudier et al. 1989, 1991; Watson et al. 1990; Deloulme et al. 1991; Huang et al. 1993; Paudel et al. 1993; Gerendasy et al. 1994) and interaction with phosphatidic acid (Domínguez-González et al. 2007). When Ca^2+^ levels reach a certain threshold inside the cell, Nrgn is phosphorylated and releases calmodulin, which can then interact with other proteins (Baudier et al. 1989, 1991; Watson et al. 1990; Deloulme et al. 1991; Gerendasy et al. 1994; Gerendasy and Sutcliffe 1997; Lee 2006; Li et al. 2020). The phosphorylated form of Nrgn may also have down-stream targets to be fully determined yet, such as the calmodulin dependent nitric oxide synthase (Martzen and Slemmon 1995) or G-protein coupled second messengers (Cohen et al. 1993; Watson et al. 1996).

Given Nrgn function in synaptogenesis and synaptic plasticity, it is not surprising that it has been related to various human neurological diseases and disorders, which include Alzheimer disease (Chang et al. 1997; Hellwig et al. 2015; Bereczki et al. 2016; Casaletto et al. 2017; Lista and Hampel 2017; Kvartsberg et al. 2019), Parkinson and Parkinsonian disorders (Koob et al. 2014; Selnes et al. 2017), schizophrenia (Giegling et al. 2010; Van Winkel et al. 2010; Gurung and Prata 2015; Wen et al. 2016; Zhang et al. 2019; Jin et al. 2019) and Huntington’s disease (DiFiglia 1990). In fact, Nrgn is used as a CSF biomarker for synapsis loss in Alzheimer disease (Lashley et al. 2018; Blennow and Zetterberg 2018a, b), and could be a marker for other diseases and pathological states (Yang et al. 2015; Bereczki et al. 2017). In addition, sleep deprivation has shown to decrease Nrgn levels (Rhyner et al. 1990; Neuner-Jehle et al. 1995), which again could indicate a role of Nrgn in synaptic plasticity.

It is also very likely that Nrgn is crucial in the development of certain areas and circuits of the brain. Neurogranin genomic region contains regulatory elements for retinoic acid and steroid hormone receptors (Iñiguez et al. 1994; Enderlin et al. 1997; Husson et al. 2003, 2004; Féart et al. 2005; Buaud et al. 2010), as well as binding domains for different transcription factors (Iñiguez et al. 1994; Martínez de Arrieta et al. 1997; Sato et al. 1995). Several studies have suggested a cell specific regulation of Nrgn expression by thyroid hormones (Muñoz et al. 1991; Iñiguez et al. 1992, 1993, 1996), likely through thyroid responsive elements within the Nrgn first intron (Martínez de Arrieta et al. 1999; Morte et al. 1999).

Despite most likely having key roles during brain development, only a couple of studies have analyzed Nrgn distribution during development in the rat (Gerendasy et al. 1994; Álvarez-Bolado et al. 1996) and the mouse olfactory bulb (Gribaudo et al. 2012). In the adult, Nrgn brain distribution was studied in the rat (Represa et al. 1990; Watson et al. 1990, 1992; Neuner-Jehle et al. 1996; Houben et al. 2000; Singec et al. 2003), mouse (Singec et al. 2003), three species of monkey (*Cercopithecus aetiops* by Singec et al. 2003, and *Macaca fascicularis and M. nemestrina* by Guadaño-Ferraz et al. 2005), adult zebra finches (Clayton et al. 2009) and recently in the adult zebrafish (Alba-González et al. 2022). In adult zebrafish, we previously showed by Western blot of brain protein extracts the presence of three Nrgn-immunoreactive peptide bands with MW corresponding to those of peptides in mouse brain extracts, validating this antibody for zebrafish brain studies (Alba-González et al. 2022). These three proteins are coded in zebrafish by two paralog neurogranin genes, *nrgna* and *nrgnb*, but distinction of cells expressing one or other of these was not studied and thus the immunoreactivity was named as neurogranin-like. The study of Nrgn-like expression along development (present results) in comparison with those of the adult stage (Alba-González et al. 2022) provides new neuroanatomical data for a more precise topological location of nuclei and tracts in early postembryonic stages in zebrafish. In addition, given the growing use of zebrafish as a model in neurobiology and the availability of tools in this species (Key and Devine 2003; Friedrich et al. 2010; Wyatt et al. 2015; Adams and Kafaligonul 2018; Vanwalleghem et al. 2018; Bao et al. 2019; Zakowski 2020), we believe our study also sets the basis for future work using zebrafish to tackle the Nrgn roles in health and disease.

## Materials and methods

### Animal maintenance and embryo collection

Wild-type zebrafish adults (*Danio rerio*) were kept in aquaria under standard conditions of 14/10 h light/dark periods, 28.0 ± 1.0 °C, pH 7.0 ± 1.0. Water quality was monitored weekly and kept within recommended parameters (0–50 mg/L nitrate, < 1 mg/L nitrite, and < 0.2 mg/L ammonium) (see Aleström et al. 2019). Adults were fed with a mixture of decapsuled *Artemia salina* and commercial dry flake food twice a day.

For obtaining embryos and larvae, adults were transferred to mating tanks in a 2:1 ratio (female: male). The next morning, fertilized eggs were collected in Petri dishes and maintained at 28.0 ± 1.0 °C in an incubator until their use.

### Neurogranin immunocytochemistry

#### Samples analyzed

Various embryonic and larval stages were analyzed, these included 1 day post-fertilization (dpf), 2 dpf, 3 dpf, 5 dpf, 5.5 dpf, 6 dpf, 16 dpf [L1 stage following Singleman and Holtzman 2014] and 21 dpf [advanced L1, Singleman and Holtzman 2014] stages.

Embryos and larvae were euthanized by tricaine methanesulfonate (MS222; Sigma, St. Louis, MO) overdose and fixed by immersion in 4% paraformaldehyde (PFA) in 0.1 M pH 7.4 phosphate buffer (PB) at room temperature. After being rinsed in saline PB (PBS), fish were transferred to PBS and kept at 4 °C until use.

#### Whole-mount immunocytochemistry

The protocol used for whole-mount immunocytochemistry in embryos and larvae was that described by Turner et al. (2014). In brief, embryos and larvae were dehydrated in 50% methanol and stored in 100% methanol at – 20 °C for at least 30 min. Samples were then rehydrated, washed 3 times (10 min each) in 0.5% Triton-X-100 in 0.1 M PBS (PBST; pH 7.4) and permeabilized with Proteinase K (Sigma-Aldrich, P2308). Then, to prevent non-specific antibody binding sites, fish were incubated with a blocking solution of 10% normal goat serum (NGS; Sigma Aldrich, G6767-19B409) in 0.5% PBST with 1% dimethyl sulfoxide (DMSO) for 1 h, and then with the primary antibodies solution (Nrgn: Rabbit Anti-Neurogranin Polyclonal Antibody; Chemicon, AB5620, Lot #3,091,673, 1:500 dilution; SV2: Mouse Anti-synaptic vesicle protein 2; DSHB AB2315387, 1:250 dilution) overnight at 4 °C. Then, fish were washed in PBST (4 times, 30 min each) and incubated with appropriate secondary antibodies (Goat Anti Rabbit IgG-Alexa Fluor 488, Invitrogen, A11008 for Nrgn immunohistochemistry and Goat Anti Rabbit IgG-Alexa Fluor 568, Invitrogen, A1104 for SV2; 1:500 dilutions) at room temperature for 1 h. After two washes in PBST (30 min each), fish immunoreacted against Nrgn antibody were counterstained with Sytox Orange Nucleic Acid Stain (Invitrogen, S11368, 1:10^4^ dilution) for 7 min at room temperature. After two washes in PBST (30 min each), fish were maintained in 50% glycerol in PB and stored at 4 °C. For imaging, embryos and larvae were transferred to 80% glycerol (30 min) and mounted in 1% low melting point agarose in 80% glycerol.

#### Immunocytochemistry in cryosections

Whole larvae (6 dpf) were kept on 30% sucrose in PB overnight at 4 °C. The next day, larvae were embedded in Tissue-Tek mounted media (Cell Path, KMA-0100-00A), frozen in methylbutane cooled in liquid nitrogen. Next, transverse sections (12–14 µm thick) were obtained using a cryostat (MICROM; HM 500 M) and collected in gelatin-coated slides. To remove autofluorescence, sections were incubated in 0.2% sodium borohydride in PBS (30 min). Sections were preincubated with normal goat serum (1 h) and then incubated with the primary antibody solution as indicated above for whole-mount immunocytochemistry (4 °C; overnight). Then, sections were washed four times in PBST (15 min each) and incubated with Goat Anti-Rabbit IgG coupled to Alexa Fluor 488 (Sigma Aldrich, A11008, 1:500 dilution) at room temperature for 1 h. After two washes with PBST (10 min each), slides were mounted using 50% glycerol in PB and maintained at 4 °C in darkness until observation.

### Imaging

Embryos and larvae were imaged using a laser scanning confocal microscope Nikon A1R equipped with Nikon Plan Fluor 10x (0.30 NA) and 20x (0.50 NA) objectives. An argon ion laser (488 nm) and a diode laser (561 nm) provided the excitation light for the fluorophores. Emission light was sequentially acquired for each channel. Confocal z-stacks were processed and analyzed using Fiji software (Schindelin et al. 2012). Red channel is shown as magenta in the figures. Sections of zebrafish larvae (6 dpf) were imaged using an Epifluorescence microscope (Nikon Eclipse 90i) coupled to an Olympus DP71 digital camera.

## Results

### Neurogranin distribution in the embryo and larva

We investigated Nrgn-like immunoreactive (Nrgn-like-ir) structures at various stages of embryonic (1–3 dpf) and postembryonic/ larval (5, 6, 16 and 21 dpf) development of zebrafish.

#### Neurogranin expression in the embryo

**Fig. 1:**
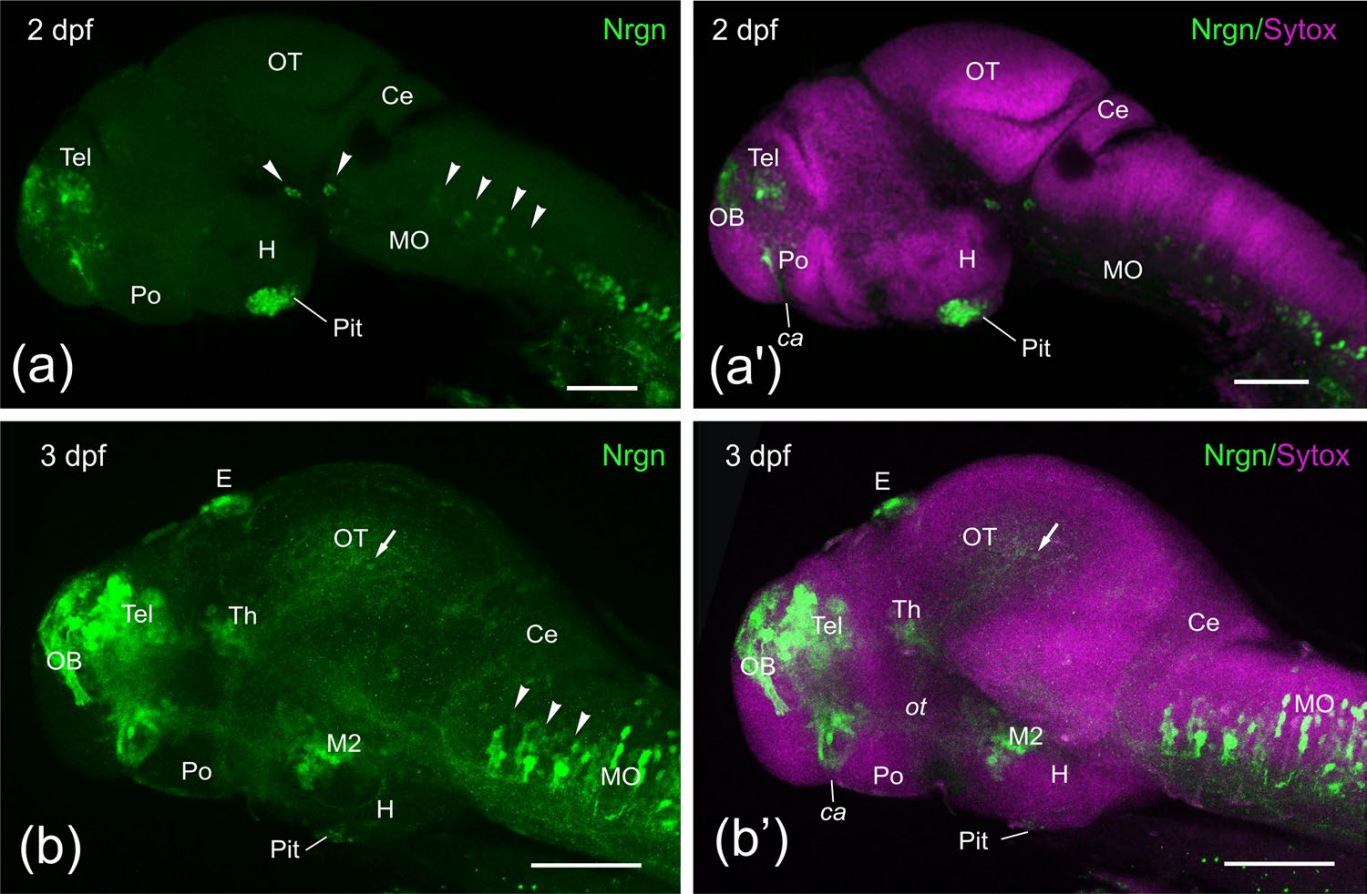
Side view of confocal projections from 2 (**a**, **a’**) and 3 (**b**, **b’**) dpf zebrafish embryos showing Neurogranin-like (Nrgn-like) immunoreaction (in green) and counterstained with Sytox Orange Nucleic Acid (in magenta). Arrowheads point to mesencephalic and rhombencephalic positive cell groups. A faintly labeled cell was also pointed in the optic tectum (arrow). Rostral is to the left and dorsal to the top. For abbreviations, see the list. Scale bars, 100 µm

No Nrgn immunoreactivity was observed in the central nervous system at 1 dpf embryos. The first Nrgn-like-ir structures appeared at 2 dpf, showing the first immunoreactive cell bodies in the pallium, the epiphyseal cluster (not shown) and the hypophysis (Fig. 1a, a’). A few sparsely distributed Nrgn-like-ir cell bodies were also observed in the preoptic region close to the anterior commissure. In addition, in the prosencephalon, immunoreactive fibers were observed in the olfactory bulb (glomeruli) and the anterior commissure. Two small compact groups of Nrgn-like-ir cell bodies were observed in the ventral region (basal plate) of the mesencephalon and the isthmus, which could represent the IIIrd (oculomotor) and IVth (trochlear) motor nuclei, respectively (see discussion). In addition, many Nrgn-like-ir cell bodies and fibers were located along the medulla oblongata and spinal cord, with some hindbrain neurons forming discrete groups in a segmental pattern (Fig. 1a, a’).

By 3 dpf, in addition to Nrgn-like-ir fibers in the olfactory glomeruli, we observed a few large Nrgn-like-ir cell bodies in the olfactory bulbs. We also observed strong immunoreactivity in cell bodies in the pallium, subpallium and preoptic region close to the anterior commissure (Fig. 1b, b’). More caudally, in addition to the expression in epiphyseal and hypophyseal cell bodies, Nrgn-like expression was also seen in the tubercular area (M2 of Mueller and Wullimann 2003) and faint Nrgn-like expression in cell bodies of the thalamus and optic tectum. Although no new immunoreactive cell groups were seen in the mesencephalic tegmentum and isthmus, an increasing number of Nrgn-like-ir cell bodies were observed in the rhombencephalic tegmentum (Fig. 1b, b’). Strongly immunostained fibers were also seen coursing the olfactory tract and the anterior commissure, while faintly labeled fibers could be observed in the supraoptic tract/ forebrain bundle (see Wilson et al. 1990; Chitnis and Kuwada 1990), the optic tract and the ventral longitudinal tract through the medulla and rostral spinal cord (Fig. 1b, b’). Only scarce tectal cell bodies showed faint Nrgn-like immunoreactivity.

#### Neurogranin expression in larvae

**Fig. 2:**
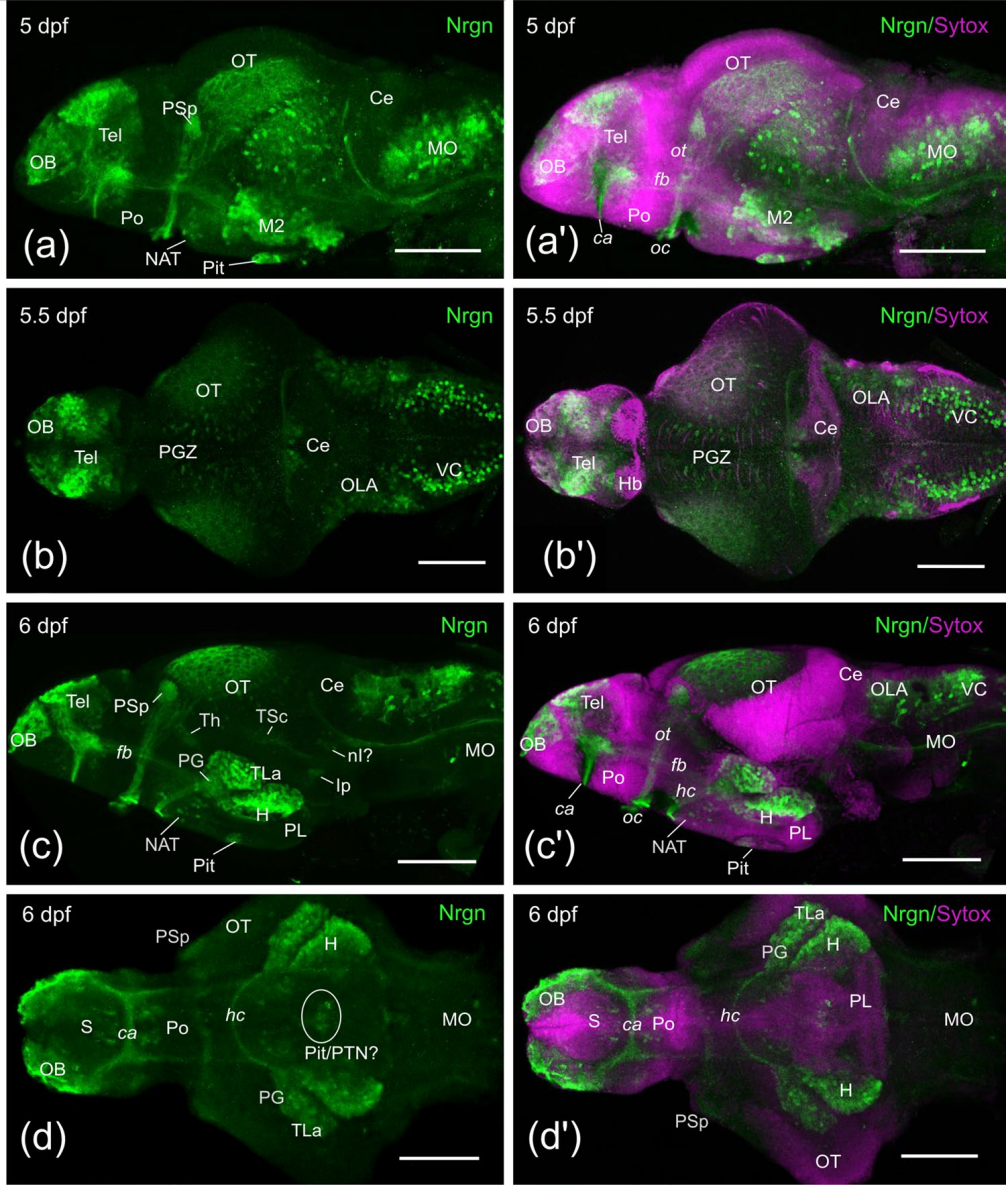
Side (**a**, **a’**, **c**, **c’**) dorsal (**b**, **b’**) and ventral (**d**, **d’**) confocal projections of zebrafish brains from 5 (**a**), 5.5 (**b**) and 6 (**c, d**) dpf larvae showing Neurogranin-like (Nrgn) immunoreaction of zebrafish brain (in green) and counterstained with Sytox Orange Nucleic Acid (in magenta). Rostral is to the left. For abbreviations, see the list. Scale bars, 100 µm

By 5 dpf, in addition to the Nrgn-like-ir structures described previously in embryos, Nrgn-like-ir cell bodies appeared in the tuberal area, hypothalamus and in the cerebellum, the later likely representing Purkinje cells of the cerebellar valvula. A significant increase in the number of Nrgn-like-ir cell bodies was noticed in the optic tectum, in the pallium and in M2 (Fig. 2a, a’, b, b’).

**Fig. 3:**
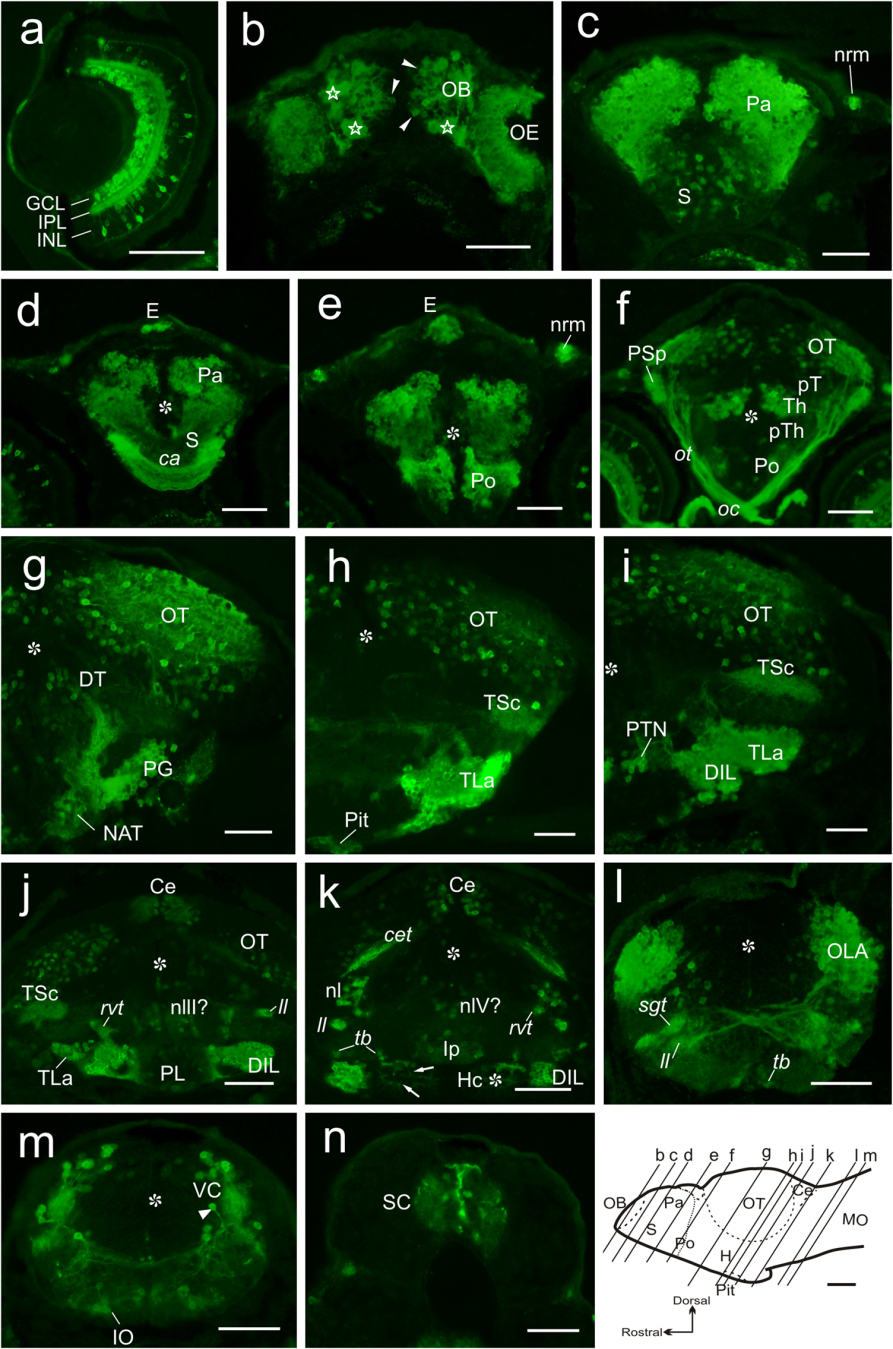
**a–n** Photomicrographs of transverse sections showing Nrgn-like immunoreaction (in green) in the retina (**a**), brain (**b–m**) and spinal cord (**n**) of a 6 dpf zebrafish larvae. Section levels are indicated in the longitudinal schema of the brain at the bottom. In **b**, outlined stars show the olfactory glomeruli. Note in **j** the slightly mismatch between both sides of the brain at mesencephalic level. Arrows in **k** point to the CSF-contacting cell processes directed towards the posterior recess of the hypothalamus. Asterisk: ventricle. For abbreviations, see the list. Scale bars, 200 µm

At 6 dpf, Nrgn-like immunoreactivity was studied both in whole-mount brain and in transverse sections from non-dissected larvae for better neuroanatomical characterization. We observed Nrgn-like immunoreactivity in the central nervous system and sensory organs. Well-developed Nrgn-like-ir cell bodies were observed in developing sensory organs as the retina, the olfactory epithelium, cranial neuromasts (Fig. 3a, c, e) and inner ear (hair cells; not shown). In the retina, Nrgn-like immunoreactivity was noticed in several populations, namely, some bipolar and amacrine cells and in most, if not all, ganglion cells. Nrgn-like-ir processes of these cells were also observed forming organized strata in the inner nuclear layer (INL) and coursing in the optic nerve and tract (Figs. 2c, c’, 3a). In the brain, we could observe a number of Nrgn-like-ir fibers in the olfactory glomeruli coming from Nrgn-like-ir receptor cells in the olfactory rosette. Double immunostaining against Nrgn and the synaptic marker SV2 confirmed that all olfactory glomeruli received Nrgn-like-ir fibers (not shown). Fibers both in the olfactory nerve and bulb were strongly labeled (Figs. 2c, c’, 3b). In the telencephalic lobes, we observed increased numbers of intensely labeled Nrgn-like-ir cell bodies in the pallium, the precommissural subpallium and the preoptic area (Fig. 3c–f). Periventricular cells of the preoptic area send projections to the ventrolateral margin and course caudally (Fig. 3e). Caudal to the anterior commissure, a large group of intensely immunolabeled cell bodies was also observed. Nrgn-like-ir cell bodies were also observed in the epiphysis, dorsal thalamus, posterior tubercle (anterior and posterior tuberal nuclei), torus lateralis, the inferior hypothalamic lobes, pituitary (probably adenohypophysis) and, more faintly stained, in the preglomerular complex and the caudal hypothalamic lobes (Figs. 2c, c’, 3d–k). Faintly immunolabeled cell bodies were also seen in the posterior lobe (Hc), showing CSF-contacting morphology around the posterior recess (Fig. 3k). Compared with previous stages, the number of Nrgn-like-ir fibers increased both in the posterior tubercle and hypothalamus. As in previous stages, Nrgn-like-ir fibers were observed in the optic tract, coursing to a conspicuous neuropil area in the pretectum (likely to be the parvocellular superficial pretectal nucleus) and also entering the optic tectum (Figs. 2c, c’, 3f). In the alar mesencephalon, the number of Nrgn-like-ir cell bodies and fibers increased in the optic tectum. Some Nrgn-like-ir cell bodies, together with fibers likely originated from the lateral lemniscus, were also observed in the torus semicircularis (Fig. 3h–j). In the mesencephalic tegmentum, Nrgn-like-ir cell bodies were observed medially close to the ventricle (Fig. 3j). In the rostral rhombencephalon, immunoreactive cell bodies were observed in the cerebellar valvula (Fig. 3h–k), and in the medial and lateral regions of the isthmic tegmentum, including the interpeduncular nucleus (Fig. 3k). Caudally, a number of Nrgn-like-ir cell bodies were observed in the octavolateralis area, the primary viscerosensory column, the reticular formation and the inferior olive (Fig. 3l–m). Some Nrgn-like-ir cell bodies were also observed in the spinal cord (Fig. 3n). In addition to the labeled fibers and cell bodies described above, we observed Nrgn-like-ir fibers in several tracts and commissures: in the anterior and horizontal commissures and in the olfactory, telencephalic, optic, tectobulbar, cerebellar and secondary gustatory/visceral tracts (Fig. 2c, c’), most of them already present in previous stages (Figs. 2, 3).

**Fig. 4:**
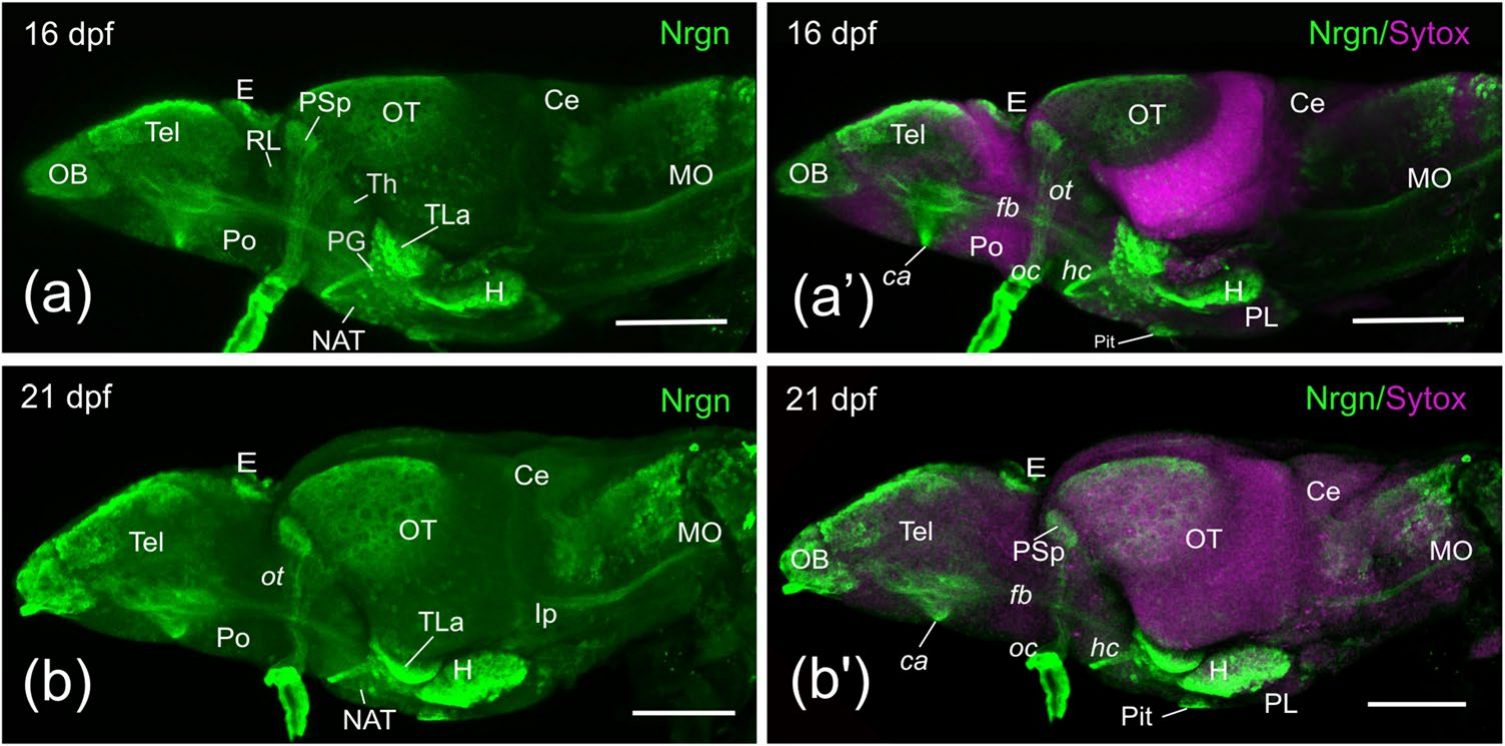
Side view of confocal projections from 16 (**a**, **a’**) and 21 (**b**, **b’**) dpf zebrafish brains showing Nrgn-like immunoreaction (in green) and counterstained with Sytox Orange Nucleic Acid (in magenta). Rostral is to the left and dorsal to the top. For abbreviations, see the list. Scale bars, 100 µm

Finally, we analyzed expression by 16 and 21 dpf in whole-mount (Fig. 4a-a’, b-b’). We observed little qualitative differences in Nrgn-like immunoreactivity compared to 6 dpf larvae. Noteworthy, the region of the pallium with cells with intense Nrgn-like expression was broader than in previous stages.

## Discussion

This study reports the appearance and changes in distribution of Nrgn-like immunoreactivity in the brain and sensory organs of zebrafish during development. Nrgn-like immunoreactivity is late appearing, since its expression starts by 2 dpf in very restricted areas of the brain but increases noticeably from 3 to 6 dpf. By 5 dpf-6 dpf, the regional expression of Nrgn-like peptides resembles that observed in the adult (Alba-González et al. 2022). We observed expression in cell bodies and fibers of specific regions of the forebrain, midbrain and hindbrain. These results expand considerably the neuronal distribution reported previously by *nrgna* mRNA in situ hybridization (Zada et al. 2014). Based on the location of the Nrgn-like-ir cells, away from the ventricular zone, it seems probable that they correspond to differentiated neurons, which would agree with observations in the cerebral cortex of rat (Represa et al. 1990; Houben et al. 2000).

Nrgn shares important biochemical similarities with other members of the calpacitin family, such as neuromodulin (Coggins et al. 1993; Gerendasy and Sutcliffe 1997) and pcp-4 (Mione et al. 2006). They share an IQ domain and, at least in mammals, they are substrates for PKC phosphorylation (Baudier et al. 1991; Deloulme et al. 1991; Watson et al. 1992; Huang et al. 2000; Kumar et al. 2013, Alba-González et al. 2022). While Nrgn seems to be mainly postsynaptic in mammals (Represa et al. 1990; Coggins et al. 1993; Watson et al. 1994; Neuner-Jehle et al. 1996), neuromodulin seems to be presynaptic and located in axons (Snipes et al. 1987; McGuire et al. 1988; Gerendasy and Sutcliffe 1997). It is noteworthy that Nrgn appears to be a marker for specific cell populations, allowing to track these populations during development (present results), as it is also the case for pcp-4, another member of the calpacitin family (Mione et al. 2006). We have observed Nrgn-like expression throughout the brain and sensory organs, which suggests that in zebrafish Nrgn-like peptides could be both pre- and post-synaptic, as shown in the rat spinal cord (Houben et al. 2000). It would be necessary a detailed study of the different Nrgn-like peptides to confirm how they relate to the synapse and their relation to neuromodulin. Below, we will discuss the main findings in specific brain and sensory systems populations.

*Olfactory system.* Present results reveal Nrgn-like expression in cells of the olfactory epithelium, as well as in the olfactory nerve and terminal fields (glomeruli) in the olfactory bulb, i.e., in the primary olfactory neurons. However, the neurons of the olfactory bulb (mitral cells, granule cells), as well as the olfactory tracts, appear to be negative in embryos/larvae, suggesting that Nrgn is presynaptic in zebrafish primary olfactory fibers. Some Nrgn-like-ir olfactory bulb neurons (likely to represent granule cells) were also observed in adults (Alba-González et al. 2022). Results in zebrafish larvae differ from those reported by Gribaudo et al. (2012) in the olfactory bulbs of developing mouse, which lack Nrgn-like immunoreactivity in olfactory fibers, but prominently express it in tufted cells and in granule cells. This suggests that Nrgn is involved in different tasks in olfactory circuits of zebrafish and mouse.

*Telencephalon*. An interesting result is the strong Nrgn-like expression in cell bodies of the primordial pallium of zebrafish larvae, which agrees with results reported with *nrgna *in situ hybridization (Zada et al. 2014). Our results of Nrgn-like expression in larval pallium correspond with that observed in some pallium regions of the adult (Alba-González et al. 2022). The larval pallium appears to originate a conspicuous Nrgn-like-ir forebrain tract that is recognizable in whole mount stained brains extending toward the hypothalamus–posterior tubercle. In addition, the pallium originates Nrgn-like-ir fibers coursing in the anterior commissure. These projections correspond with those reported in detail with DiI tracing from some pallial regions of the adult zebrafish (Yáñez et al. 2022). However, it was not possible to identify the different adult pallial regions in larvae, which precludes more detailed comparisons. The strong expression of Nrgn found in projection neurons of the zebrafish pallium reminds the distribution of Nrgn in principle neurons of various pallial areas of rodents (Álvarez-Bolado et al. 1996), although projection neurons appear morphologically much more specialized in rodents. The pallium of two oscine birds also expresses high Nrgn mRNA levels (Clayton et al. 2009).

The development of the Nrgn-like expression in zebrafish telencephalon shows some differences with that reported in the developing rat (Álvarez-Bolado et al. 1996). In the rat telencephalon, Nrgn expression starts in the primordium of the amygdala and the piriform cortex at embryonic stage 18, increasing the areas of expression on postnatal week 1, when Nrgn immunoreactivity appears in olfactory cortex, isocortex, subiculum, hippocampus, striatum (caudoputamen) and parts of the globus pallidus and septum (Álvarez-Bolado et al. 1996). In the zebrafish nervous system, Nrgn-like expression is observed from 2 dpf, before the animal hatched from the chorion and is capable of independent feeding (Kimmel et al. 1995; Strähle et al. 2012; Filosa et al. 2016). At this stage, we observe Nrgn-like expression in cell bodies of the pallium, which is similar the situation in the rat.

*Visual system.* In the retina of developing zebrafish, Nrgn-like is expressed largely in ganglion cells but also in numerous amacrine cells and some bipolar cells. Both inner and outer plexiform layers show Nrgn-like immunoreactivity, which is prominent in sublayers of the inner plexiform layer, unlike its poor expression in adults (Alba-González et al. 2022). As far as we are aware, there are no reports of distribution or development of Nrgn expression in the retina of other vertebrates. The strong Nrgn-like expression in retinal ganglion cells during development is also observed in their axons. *In toto* staining reveals Nrgn-like positivity in the optic nerve and optic tract since 3 dpf, as well as conspicuous immunoreactivity in two visual afferent fields, that is, AF7 (corresponding to the adult parvocellular superficial pretectal nucleus, PSp, located in p1) and the optic tectum. Other afferent fields of the optic pathway were less easily recognizable (for a description of the different AFs in larval zebrafish see Robles et al. 2014, and Baier and Wullimann, 2021). As reported in adults, the AF7 neuropil has no associated Nrgn-like-ir cells, nor the conspicuous ventral commissure linking the PSp of both sides (Castro et al. 2006a; Yáñez et al. 2018) was labeled. The optic tectum, as in the adult zebrafish (Alba-González et al. 2022), showed abundant Nrgn-like-ir cells, most showing their somas in the thick periventricular cell layer. Whereas the zebrafish visual system shows abundant expression of Nrgn-like early on in development, suggesting it is an important peptide for this system, the lack of data on this system in other vertebrates precludes further comparison.

*Diencephalon and segmental distribution of Nrgn*. The diencephalon of vertebrates, including zebrafish, consists of three prosomeres (p1–p3), from caudal to rostral, with several alar and basal plate derivatives (Puelles and Rubenstein 1993; Wullimann and Puelles 1999; Hauptmann et al. 2002; Mueller, 2012). Convenient sections as that presented in Fig. 3f, show that the three prosomeres are different with respect to Nrgn-like expression in neuronal populations. The most conspicuous Nrgn-like expressing population corresponds to that of the thalamus (alar region of p2), whereas alar p3 (prethalamus) and p1 (pretectum) lack similar populations. The neurons of the zebrafish thalamus are mostly glutamatergic and at least some nuclei project to the pallium (Mueller 2012; Yáñez et al. 2022). Unlike the thalamus, the most dorsal region of p2 (the habenula) neither shows Nrgn-like-ir neurons, nor the habenular commissure shows Nrgn-like-ir fibers. The alar region of p1 (pretectum) mostly consists of GABAergic populations (Mueller et al. 2006; Mueller 2012). Pretectal neurons do not express Nrgn, nor Nrgn-like-ir fibers are observed in the posterior commissure, the most conspicuous of the brain dorsal commissures. The Nrgn-like-ir cells of the epiphysis probably are not projection neurons, because fibers of the epiphysis tract were not labeled. This thin but conspicuous tract appears very early in zebrafish development (Wilson et al. 1990).

*Hypothalamus*. The hypothalamus is considered the ventral region of the secondary prosencephalon in neuromeric models of the brain (Puelles and Rubenstein 1993; Affaticati et al. 2015). In zebrafish and other teleosts, its caudal (ventral) region evaginates to form lateral and posterior recesses of the infundibulum around which become organized the hypothalamic lobes (inferior and posterior), an impar saccus vasculosus (in some teleosts but not in zebrafish), as well as outstanding groups of neurons, some protruding laterally or caudally (preglomerular complex, torus lateralis, diffuse nucleus, mammillary nucleus). The origin of these neuronal populations of teleosts is complex, because they originate from the primordial hypothalamic walls and from cells migrating tangentially from posterior tubercular/midbrain regions (Bergqvist 1932; Corujo and Anadón 1990; Bloch et al. 2019, 2020). The origin of some migrating populations was recently traced in transgenic zebrafish to the midbrain (Bloch et al. 2019, 2020), and these migrating cells travel during several days before reaching its hypothalamic location. Present results reveal that the conspicuous hypothalamic populations of Nrgn-like-ir cells at 5–6 dpf appeared by 3 dpf, i.e., before the arrival to the torus lateralis and hypothalamic lobes of the midbrain migrating population. Moreover, these Nrgn-like-ir populations of the torus lateralis and diffuse nucleus give rise to a conspicuous Nrgn-like-ir tract (tract of the horizontal commissure) that decussates ventrally and caudally to the optic chiasm in the horizontal commissure, which is characteristic of teleost fishes. Instead, the preglomerular population originated in the midbrain projects ipsilaterally to the pallium without forming any commissure (see Fig. 2c, d in Bloch et al. 2020). This suggests that these Nrgn-like-ir embryonic populations originate from the hypothalamic primordia, mixing with those tangentially migrating from the midbrain demonstrated by Bloch et al. (2019, 2020).

*Hindbrain*. The expression of Nrgn-like immunoreactivity during hindbrain development shows a segmental pattern of the first positive neurons. This is clearly appreciable in 2 dpf and 3 dpf embryos, where small groups of Nrgn-like-ir cell bodies can be ascribed to rhombomeres 2 to 6. Segmental patterns of early hindbrain populations have been reported for reticulospinal and motoneurons (Metcalfe et al. 1986; Hanneman et al. 1988), which develop much earlier than the Nrgn-like-ir cells. In 5 dpf and 6 dpf larvae the number of Nrgn-like-ir cells increased considerably, and the segmental groups have coalesced longitudinally forming two partially overlapped columns in the dorsolateral hindbrain. Careful observation of these columns and comparison with topographical expression of key markers in 6 dpf brain reveals that Phoxb2 and VGlut expressions in the zebrafish brain browser application (http://vis.arc.vt.edu/projects/zbb/) (Marquart et al. 2015) allows to easily distinguish between the octavolateralis column (OLA) (VGlut + , Phoxb2-) and the viscerosensory column (VGlut-, Phoxb2 +) at dorsal hindbrain regions. The dorsolateral Nrgn-like-ir population corresponding to the viscerosensory column extends between r4 (rhombomere 4) and the obex (caudal hindbrain, where both sides fuse) and that of the octavolateralis column (OLA) extends between r2 and r4–r5. In its rostral level the viscerosensory column becomes thinner and shifts to locate medial to the OLA. The Nrgn-like-ir viscerosensory column coincides with a cellular band that expresses Phoxb2 (Coppola et al. 2012), whereas this marker is not expressed in the OLA. In adult zebrafish, the viscerosensory column shows three lobes (facial, glossopharyngeal and vagal sensory lobes), two of them conspicuous (Wullimann et al. 1996; Castro et al. 2006b; Yáñez et al. 2017). In larvae, a conspicuous Nrgn-like-ir ipsilateral ascending tract ending in the cerebellar region can be identified as the secondary gustatory tract projecting to the secondary gustatory nucleus, as reported with tract tracing in adults (Yáñez et al. 2017), although the cells of this nucleus were Nrgn negative.

As indicated above, combination of Phoxb2 and VGlut expressions in the Z Brain Browser application (http://vis.arc.vt.edu/projects/zbb/) (Marquart et al. 2015) can be used for easily distinguishing between the Nrgn-like-ir OLA (VGlut + , Phoxb2-) and the Nrgn-like-ir viscerosensory column (VGlut- and Phoxb2 +) at dorsal hindbrain regions. The location of the hindbrain area responsible to auditory stimuli in 6 dpf larvae (Constantin et al. 2020) appears to match with that the OLA reported here. In the case of the OLA, in transverse hindbrain sections it can be appreciated how the Nrgn-like-ir OLA gives rise to abundant arcuate fibers crossing the midline and coursing in the lateral lemniscus toward the midbrain (maybe torus semicircularis), i.e., in the reported main efferent pathway of the OLA (Vanwalleghem et al. 2017; Constantin et al. 2020). With regards the developing cerebellum, Nrgn-like expression is low in most cerebellar regions in contrast with that observed in the OLA. The zebrafish cerebellum has been molecularly characterized in adults and development by Bae et al. (2009). As in the case of the viscerosensory column, we have not found reports of Nrgn distribution in these hindbrain regions of other vertebrates, which precludes comparative comparisons.

*Possible roles of Nrgn during brain development*. The fact that Nrgn is expressed in the brain from early stages in zebrafish (present results) and rat (Gerendasy et al. 1994; Álvarez-Bolado et al. 1996) suggests it plays some roles during development, maybe in axonal growth, plasticity and synaptogenesis. In zebrafish, pathfinding and synaptogenesis has already started by 2 dpf, and thus Nrgn could have a role in these processes under regulation by thyroid hormones and other signals (Muñoz et al. 1991; Iñiguez et al. 1992, 1993, 1994, 1996; Enderlin et al. 1997; Husson et al. 2003, 2004; Féart et al. 2005; Buaud et al. 2010). In fact, zebrafish larva shows a 46% increase in *nrgna* transcript after T3 thyroid hormone administration (Zada et al. 2014), which points to thyroid regulation of Nrgn expression during development, as shown in mammals (Iñiguez et al. 1992, 1996; Piosik et al. 1995; Martínez de Arrieta et al. 1999; Dowling and Zoeller 2000; Zoeller et al. 2005; Stepien and Huttner 2019). In the rat brain, a peak of Nrgn expression has been described between postnatal days 10 and 20 (Represa et al. 1990; Watson et al. 1990; Álvarez-Bolado et al, 1996), which may be coupled with a peak in synaptogenesis. Our data does not allow analyzing differences in expression levels between different stages, as levels of confocal signal (gain) was adjusted individually for every imaged specimen, so levels of expression between stages are not comparable. We did observe a sustained expression of Nrgn-like throughout development, without transient expression in any area or cell type, i.e., once Nrgn-like expression is observed in one area it is maintained in development and in the adult (Alba-González et al. 2022). This is similar to results in the mammalian brain (Represa et al. 1990; Watson et al. 1990; Álvarez-Bolado et al. 1996; Guadaño-Ferraz et al. 2005), because loss of expression at a given area or cell type has only been reported in the mouse olfactory bulb (Gribaudo et al. 2012).

*Comparison with the expression of pcp4a in zebrafish*. It is worth noting that our results show that Nrgn is a marker for specific neuronal populations, allowing tracing these populations during development. This is also the case for pcp4a, another member of the calpacitin family studied by in situ hybridization in developing and adult zebrafish (Mione et al. 2006). Some parallelisms can be noted in the distribution of pcp4a mRNA (Mione et al. 2006) and Nrgn-like peptides (present results). In both cases, first expression is observed in differentiating neurons, and not in proliferating zones. Some neuronal populations show expression of both pcp4a and Nrgn in development, but there are others expressing one or the other, suggesting only partial codistribution. Among populations expressing both substances, the most outstanding are the retinal ganglion cells. Other areas showing possible colocalization or codistribution of both pcp4a and Nrgn are the pallium, the dorsal thalamus, the optic tectum, the torus semicircularis, cerebellum and the viscerosensory area (Mione et al. 2006; present results). The dorsal habenula is pcp4a positive but Nrgn negative, and the same appears to occur with the preglomerular complex, pseudoglomerular nucleus, mammillary bodies and reticulospinal neurons (Mione et al. 2006; present results). Among the Nrgn-like-ir populations that are largely pcp4a negative during development, it is worth mentioning the amacrine and bipolar cells of the retina, and the inferior lobes. Thus, although both Nrgn and pcp4a interact with calmodulin in neurons, facilitating adaptation, they appear to be selectively used by some centers. Further comparison of pcp4a and Nrgn-like expression in developing zebrafish is precluded because of the different nature of the methods used by Mione et al. (2006) and in the present study. For instance, whereas Nrgn immunohistochemistry allowed studying tracts and neuropil regions, the techniques applied in Mione et al. (2006) did not allow showing these important anatomical features. Further studies should address in detail possible relations between pcp4a and Nrgn, as well as other calpacitins, in developing zebrafish neurons.

*Final consideration*. There are many aspects of Nrgn function in the adult brain and during development yet to be clarified, research in which zebrafish will most certainly contribute, given the number of tools available to work in these species. Study of Nrgn function has attracted little attention so far, but this may change soon, as a Nrgn mutant has been generated as part of a project investigating the phenotype of zebrafish carrying mutations in human schizophrenia-associated genes (Thyme et al. 2019). This project highlights the potential implication of Nrgn and zebrafish in understanding human disease.

## Conclusions

Our study of Nrgn-like immunoreactivity in neural tissues during the development of the zebrafish reveals positive cells in both sensory organs of the head (retina, olfactory rosette, neuromasts) and in the brain. Nrgn-like expression appears late in the positive populations, suggesting that it is expressed in cells that are differentiated functionally. Main Nrgn-like-ir populations in the brain were observed in the pallium, hypothalamic lobes, thalamus, optic tectum, octavolateralis area and viscerosensory column, suggesting close relationship of Nrgn with processing sensory information, probably contributing to adaptative responses in larval stages. The restriction of its expression to specific neuronal populations, combined with observations *in toto*, allowed to use Nrgn-like immunoreactivity to reveal the origin of some tracts and commissures.

## Data Availability

The data that support the findings of this study are available from the corresponding authors upon reasonable request.

## Abbreviations

*ca*: Anterior commissure
Ce: Cerebellum
*cet*: Cerebellar tract
DiL: Diffuse nucleus of the inferior hypothalamic lobe
DT: Dorsal thalamus
E: Epiphysis
*fb*: Forebrain bundle
GCL: Retinal ganglion cell layer
H: Hypothalamus
Hb: Habenulae
Hc: Caudal zone of periventricular hypothalamus
*hc*: Horizontal commissure
INL: Retinal inner nuclear layer
IO: Inferior olive
Ip: Interpeduncular nucleus
IPL: Retinal interplexiform layer
*ll*: Lateral lemniscus
M2: Posterior tubercular area
MO: Medulla oblongata
NAT: Anterior tuberal nucleus
nI: Nucleus isthmi
nIII: Oculomotor nucleus
nIV: Trochlear nucleus
nrm: Neuromasts
OB: Olfactory bulb
*oc*: Optic chiasm
OE: Olfactory epithelium
OLA: Octavolateralis area
OT: Optic tectum
*ot*: Optic tract
Pa: Pallium
PG: Preglomerular complex
PGZ: Periventricular grey zone
Pit: Pituitary
PL: Posterior lobe
Po: Preoptic area
PSp: Parvocellular superficial pretectal nucleus
pT: Pretectum
pTh: Prethalamus
PTN: Posterior tuberal nucleus
RL: Rostrolateral nucleus
*rvt*: Rostral visceral tract (Yáñez et al. 2017)
S: Subpallium
SC: Spinal cord
*sgt*: Secondary gustatory tract
*tb*: Tectobulbar tract
Tel: Telencephalic lobes
Th: Thalamus
TLa: Torus lateralis
TSc: Torus semicircularis
VC: Viscerosensory column
